# Reactance to Transgressors: Why Authorities Deliver Harsher Penalties When the Social Context Elicits Expectations of Leniency

**DOI:** 10.3389/fpsyg.2016.00550

**Published:** 2016-05-09

**Authors:** Celia Moore, Lamar Pierce

**Affiliations:** ^1^Organisational Behaviour, London Business SchoolLondon, UK; ^2^Olin Business School, Washington University in St. Louis, St. LouisMO, USA

**Keywords:** ethics, transgressions, punishment, leniency, psychological reactance, drunk driving

## Abstract

This paper combines experimental and field data to examine how authorities with discretion over how rules are enforced penalize transgressors when the social context of the transgression elicits expectations of leniency. Specifically, we test how transgressors are punished when it is their birthday: a day that triggers expectations of lenient treatment. First, in three scenario studies we explore individuals’ intuitions about how they would behave and expect to be treated if they transgressed on their birthdays, as well as how they would imagine penalizing a birthday transgressor. Second, using more than 134,000 arrest records for drunk driving in Washington State, we establish that police officers penalize drivers more harshly when it is their birthday. Then, in a lab experiment in which we grant participants discretion over enforcing the rules of an essay-writing contest, we test psychological reactance toward transgressors who make their birthday salient, even subtly, as the mechanism behind this increased stringency. We rule out several alternative explanations for this effect, including public safety concerns, negative affect and overcompensation for bias. We conclude with a discussion of the theoretical and practical implications of our findings for the literatures on punishment, rule-breaking, and legal transgressions.

## Introduction

Scholars have theorized about when and how to punish individuals who transgress laws, rules, or regulations (Arvey and Jones, 1985; Butterfield et al., 1996), examined the consequences of punishment, in particular its impact on the attitudes and subsequent behavior of the punished individual (Ball et al., 1994; Podsakoff et al., 2006), and looked at how formal systems or written policies and procedures shape punishment decisions (Beyer and Trice, 1984). Other work has explored what motivates individuals to punish, and how their judgments of appropriate punishment change as a function of the seriousness of the offense and the intentions of the offender (Robinson and Darley, 1997; Carlsmith et al., 2002; Carlsmith and Darley, 2008). However, this body of work has overlooked how the social context of a transgression influences punishment decisions.

In this paper, we examine how transgressions are penalized when they occur in a social context that elicits expectations of leniency. Transgressions that occur in a social context in which the transgressor expects leniency put authorities with the discretion over punishing them into a difficult bind, needing to balance the motivation to meet the expectations elicited by the social context with the competing motivation to punish fairly and effectively. We argue that although transgressors may believe that transgressing in a social context that elicits expectations of leniency will lead to lighter penalties, this belief is misguided. Instead, we argue that—contrary to intuition—when authorities have a responsibility to enforce rules, but face a conflicting motivation to be lenient, they resolve this conflict in favor of harsher penalties rather than in favor of increased leniency.

Our research makes several theoretical contributions. First, we contribute to existing research on punishment by exploring how the social context of transgressions influences punishment decisions. Second, we extend our understanding of how individuals manage conflicting motivations when they have discretion over penalties. This is important because situational factors that trigger expectations of preferential treatment are pervasive, but many do not justify leniency in punishment decisions. Third, our work extends the literature on bias in punishment decisions by shifting the focus from discrimination on the basis of demographic characteristics such as race or gender to a focus on how people manage competing motivations to act. Ultimately, this work informs our understanding of the challenges in exercising discretion fairly and effectively (Kadish, 1961; Sherman, 1984; Pierce and Snyder, 2012).

## Discretion in Punishment

While laws and regulations provide guidelines for how to punish transgressions, individuals (e.g., managers, judges, or police officers), and groups (e.g., panels, boards, or juries) typically have discretion regarding whether and how much to punish those who transgress. Discretion over arrests (Reiss, 1984) and prosecutions (LaFave, 1970) is a central element of most legal and regulatory regimes because it allows authorities to consider an act’s potential mitigating circumstances. However, discretion can also have negative consequences, including threats to due process (Kadish, 1961), abuses of power (Vorenberg, 1976), and biased treatment of individuals (Smith and Alpert, 2007). Whether discretion can be exercised appropriately is important, as exercising it poorly can delegitimize the work of authorities and undermine the equity and the efficacy of enforcement systems.

In the United States, the risk that discretion in punishment leads to unfair treatment of certain demographic groups has led to a number of high-profile initiatives to understand the extent and implications of these biases (e.g., Police Executive Research Forum, 2001; Lovrich et al., 2007). Most of these efforts have focused on ensuring that those with discretion over punishment do not treat transgressors differentially based on their demographic characteristics. Meanwhile, public discourse has neglected other potential biases affecting punishment decisions. Here, we suggest that individuals have difficulty managing situations where their formal responsibility to punish conflicts with a situational contingency that leads to expectations that a transgressor will be treated leniently.

### Expectations of Leniency vs. an Obligation to Punish

One body of work that focuses directly on how we are motivated to punish is the literature on “just deserts” (Carlsmith et al., 2002; Carlsmith and Darley, 2008). This research explores how individuals shift their view of appropriate penalties for a crime depending on characteristics of the act and its perpetrators. The fundamental finding of this literature is that individuals are motivated to punish crimes in proportion to the magnitude of the harm they have caused and the availability of extenuating circumstances for the act. Though some aspects of the social context in which an offense occurs create extenuating circumstances, the only contextual factors that have been studied as motivations for leniency in punishment decisions are those that are directly relevant to attributions of blame or responsibility, such as whether the act was accidental (Carlsmith et al., 2002).

However, transgressions always occur in a broader social context. Elements of this broader social context may motivate expectations of leniency, but are arguably unrelated to the crime itself. Some elements of the social context create legitimate reasons to treat transgressors gently. For example, there is a norm of treating young people more leniently than adults when they transgress rules, as there are good arguments for why culpability is impaired before reaching maturity (Steinberg and Scott, 2003). Similarly, victims of long-term domestic violence and abuse are often punished with leniency, as their violent crimes are considered more justifiable (Ammons, 1994). These aspects of the social context that motivate expectations of leniency are often enshrined in legal structures, as evidenced in different sentencing guidelines for juvenile offenders, or special legal exceptions in the case of battered women.

Other aspects of the social context that motivate people to treat transgressors more leniently are less legitimate. Social norms of deference to authority (Milgram, 1974; Cialdini, 2009) result in higher-status individuals receiving more lenient penalties than lower-status individuals, at least for minor-to-moderate transgressions (Karelaia and Keck, 2012). The attractiveness of a perpetrator also appears to motivate more lenient punishment, even though how attractive someone is has nothing to do with how a transgression ought to be penalized (Sigall and Ostrove, 1975; Piehl, 1977; Stewart, 1985). These aspects of the overarching context of the crime clearly motivate more lenient treatment of these offenders, but there are strong arguments against using them as reasons for leniency.

“Special days” such as birthdays also elicit expectations of preferential or favorable treatment that may extend to expectations of leniency in the context of transgressions. Birthdays are part of a larger class of days that have social or religious significance (e.g., Christmas, Yom Kippur) and are associated with strong norms of helping, kindness, and forgiveness. These days affect pro-social behavior such as charitable contributions (Jiobu and Knowles, 1974; Waldfogel, 1993). Birthdays specifically elicit expectations of favorable treatment, particularly for the individual whose birthday it is (Greene et al., 1987). The fact that many retailers offer free goods or services on individuals’ birthdays, from pints of beer to pizzas, likely reinforces these expectations^1^

It is not a big leap to suggest that the expectation that individuals should receive special treatment on their birthdays will even extend to expectations of leniency when important rules are broken. On December 2, 2012, the rapper known as “The Game” was pulled over by Los Angeles Police because the car he was driving had invalid license plates. A celebrity website recounted that although the car was unregistered, the police released him and did not tow his car “because it was his birthday.”^2^ The reason the website reported for the leniency shown by the officers – “because it was his birthday” – suggests that the strength of the social cues to treat people favorable treatment on this day will extend to include leniency for legal transgressions.

In this paper, we focus on the social context of birthdays as a situational cue that motivates leniency for two reasons. First, even though this norm should be irrelevant in punishment decisions, for someone with the responsibility to penalize transgressors, a birthday elicits a competing motivation to treat that particular individual with leniency. Second, birthdays are ideal for studying the effect of the social context in non-experimental field settings because they are randomly distributed in the population: in most contexts, authorities that apprehend transgressors do not and could not have known it was transgressor’s birthday before apprehending them. This means that birthday and non-birthday transgressors are randomly assigned to punishers, reducing endogeneity concerns about selection bias and causality when identifying variation in punishment decisions using data from the field.

Hypothesis 1a. Individuals will expect more lenient punishment in a social context associated with preferential treatment (i.e., individuals will predict that transgressors will be punished more leniently if it is their birthday).

#### The Transgressor’s Perspective

If someone is caught transgressing a rule on their birthday, they have a choice between making that fact salient or not. There are several reasons why transgressors are likely to make salient a relevant fact that may motivate lenient treatment of them (“But it’s my birthday!”). Individuals tend to volunteer reasons for their misbehavior in order to save face and reduce embarrassment (Goffman, 1955; Keltner and Anderson, 2000). Individuals also use available reasons in an effort to excuse their misbehavior and to transform how responsibility for actions are understood (Snyder et al., 1983). Given the strong expectation of favorable treatment associated with birthdays, we predict the following:

Hypothesis 1b. If an individual transgresses on their birthday, they will volunteer that fact in an effort to secure leniency.

#### The Authority’s Perspective

The person in a position of authority needs to manage competing motivations to be lenient and to punish in a fair and effective way. Several reasons suggest that these competing motivations might be resolved in favor of increased leniency. Of course, the authority might try to ignore the expectation of leniency and punish transgressions as if this expectation did not exist. However, as prior research demonstrates, individuals often proceed rather automatically to enact scripted cues to behave in certain ways, even when the cue is completely irrelevant to the behavior it mindlessly triggers (Langer et al., 1978). Authority figures may be more lenient in this situation because they mindlessly enact this scripted cue.

Alternatively, it might be uncomfortable for an authority to behave counter to this expectation, particularly if they have the discretion over the transgressor’s punishment. Extensive research shows that people tend to behave consistently with what is perceived to be the normative expectation in the situation, particularly when those norms are made salient (Reno et al., 1993). Thus, if an authority figure has discretion over the transgressor’s punishment, their motivation to comply with social norms would also suggest they will treat the transgressor more leniently.

Such a prediction, however, ignores important psychological mechanisms involving the interaction between the transgressor and the authority. If the transgressor draws attention to his birthday, even subtly (as they are likely to, in an effort to capitalize on the expectation they have that this will lead to lenient treatment), this may shift the decision process of the authority. Although authorities may be indifferent or even positively inclined to treat a birthday transgressor with leniency, they may be particularly sensitive to perceptions that leniency is being solicited and react negatively to them. Consistent with the drive to punish in a fair and effective way, authorities may penalize transgressions more stringently when the social context cues expectations of leniency, because they will react negatively to any perception that their leniency is being solicited.

There is sound theoretical basis for such a prediction of stringency in psychological reactance theory (Brehm, 1966; Miron and Brehm, 2006). Three factors support the argument that authorities will experience psychological reactance when their obligation to punish occurs in a social context associated with expectations of leniency. First, individuals react strongly to sources of external influence they perceive as restricting their behavioral autonomy. In situations where individuals have the discretion to help others, any perception that their benevolence is not freely volunteered will trigger reactance. The level of reactance triggered is magnified to the extent that the freedom “not to help” is important (Brehm and Brehm, 1981, p. 171). Since those charged with penalizing transgressions are strongly motivated not to help those whom they are obligated to punish, any perception that an individual is attempting to capitalize on aspects of the social context that trigger expectations of leniency will elicit reactance.

Second, reactance effects increase when requests for help appear inappropriate or illegitimate (Berkowitz, 1969, 1973). For example, Berkowitz (1969) found that individuals were less likely to help when they felt they were being coerced, and Gibbons and Wicklund (1982) suggested that acts of spontaneous helping require a situational cue to help that is both salient and legitimate. In other words, although a birthday might be a legitimate reason to let someone choose a restaurant that no one else likes, a birthday is an inappropriate reason to excuse him from the consequences of transgressing rules or laws. Thus, making a transgressor’s birthday salient in the context of a transgression will likely elicit reactance.

Finally, when authorities have an obligation to penalize transgressions, they are motivated to ensure that the punishment is fair and appropriate. Brehm and Cole (1966) found that requests for help were counterproductive and elicited reactance when target participants were told to evaluate the person requesting help accurately. A motivation to treat someone fairly and appropriately is similar to a motivation to evaluate someone accurately. Thus, we argue that even a subtle perception that a transgressor is trying to use aspects of her social context to solicit more lenient treatment will trigger psychological reactance – because this will lead the authority to perceive that their freedom to exercise that discretion is being threatened.

Hypothesis 2. When a transgression occurs in a social context that elicits expectations of leniency, individuals with discretion over penalizing that transgression will react negatively to any action that makes this expectation salient (such as mentioning the fact that it is a transgressor’s birthday).

We argue that when individuals perceive that someone is demanding something from them, whether the demand is explicit (actively soliciting leniency) or implicit (making salient an element of the social context that creates an expectation of leniency), they will experience the demand as a threat to their autonomy, and become less inclined to do it (Berkowitz, 1973). Regardless of the source of the perceived autonomy threat (e.g., choice restrictions, influence from norms, suggestions), individuals are motivated to counter the restriction and take actions to reestablish the threatened autonomy (Brehm, 1966). This reactance can operate below conscious awareness or intent (Chartrand et al., 2007) but can be extreme enough to cause a behavioral backlash in which the individual does the opposite of what she believes she is being asked to do (Fitzsimons and Lehmann, 2004). Thus, we propose:

Hypothesis 3a. Transgressions will be penalized more stringently when the social context in which the transgression occurs creates expectations of leniency (such as when it is the transgressor’s birthday).

Hypothesis 3b. The increased stringency with which transgressions will be penalized when the social context elicits expectations of leniency will be mediated by psychological reactance.

## Overview of Studies

We now present six studies that test this argument using multiple methods and data sources. First, a series of scenario studies establishes that birthdays do represent a social context in which transgressors expect leniency, even though when individuals imagine themselves as authority figures with discretion over punishment decisions, they report higher levels of psychological reactance toward birthday offenders. Second, using 9 years of DUI (Driving Under the Influence) arrest records in the state of Washington (over 134,000 arrest records), we show that police officers punish marginal offenders more stringently on their birthdays than on other days. In a series of robustness checks, we show that it is unlikely that these results are explained by substantive differences in intoxication or public safety risk, but are instead likely based on the discretionary decisions of officers. Third, in a lab experiment in which we vary the birthday status of individuals who have transgressed rules, we demonstrate that individuals treat transgressors more stringently on their birthdays as a function of the psychological reactance triggered by the birthday status of the transgressor. A final study, using a similar experimental paradigm in the lab, rules out overcompensation for bias as the mechanism behind our effects.

### Studies 1a–c: Individuals’ Intuitions about Birthday Transgressions

We ran three studies, in separate online samples, to explore individuals’ intuitions about whether they would expect to be treated leniently if they were pulled over for drunk on their birthday (Study 1a), whether they would volunteer that it was their birthday if they happened to be pulled over by a police officer on that day (Study 1b), and what they believe they would do themselves if they had discretion over penalizing a marginally drunk driver on their birthday (Study 1c). Together, our aim was to build a picture of what might occur in an actual interaction between a transgressor and an authority with discretion over penalizing the transgression on the transgressor’s birthday. These studies were conducted on Amazon Turk, an online labor market where ‘requesters’ can post short tasks for ‘workers’ to complete for a small fee. Studies have found that data collected through Amazon Turk are of comparable quality to data collected through more traditional methods (Buhrmester et al., 2011; Goodman et al., 2013; Hauser and Schwarz, 2015).

#### Study 1a

The first solicited individuals’ intuitions about how they expect they would be treated if they made their birthday salient in the context of transgressing. We predicted that their intuition would be that they would be treated more leniently on their birthdays.

##### Participants and procedure

We paid 306 participants (60% male; *M*_age_ = 32 years, *SD* = 11.1) $0.50 to respond to a scenario. There were three conditions in the experiment. In a control condition, nothing about a birthday was mentioned. In two additional conditions, we asked participants to imagine it was their birthday, which they either mentioned to the officer [**birthday-mentioned**] or not [**birthday-not-mentioned**]. We included a birthday-not-mentioned condition to understand whether individuals’ intuitions about their treatment would depend on whether or not they made their birthday salient to the officer. The scenario read:

*Imagine you are driving home after an evening out with friends. You had a couple of drinks but you feel OK driving home by yourself. As you are driving, the local police stop you. The officer notices a faint smell of alcohol, though you are speaking clearly. To be safe, they ask you to take a breathalyzer test. It turns out that your blood alcohol content is 0.075%. The legal limit is 0.08%. Since your BAC is just below the legal limit, the local cops have discretion about how to proceed. While they are not required to arrest you, they may do so and test you again at the police station. They may also choose to release you with a warning.* [**birthday-mentioned:** *Imagine also that it is your birthday. You* [**birthday-not-mentioned: *do not***] *mention this to the police officer who has stopped you.*]

Participants were then asked to make a forced choice prediction about whether the officer would arrest them or release them with a warning.

##### Results

No one failed the attention check in this study, and everyone completed the main outcome measures; thus, results are reported for the whole sample. There were significant differences by condition in terms of the proportion of respondents who believed they would be arrested, χ^2^(1, *N* = 306) = 9.45, *p* = 0.009. When asked to predict what the officer would do, 25% of respondents in the birthday-mentioned condition and 35% of respondents in the birthday-not-mentioned condition believed they would be arrested, which represent more lenient treatment than the 45% of respondents in the control condition who predicted they would be arrested. Greater leniency was predicted in the two birthday conditions, compared to the control condition, χ^2^(1, *N* = 306) = 7.21, *p* = 0.007. The difference between the birthday-mentioned and birthday-not-mentioned condition was not statistically significant at conventional levels, χ^2^(1, *N* = 209) = 2.43, *p* = 0.12, although the results were consistent with greater expectations of leniency in the birthday-mentioned condition, compared to the birthday-not-mentioned condition. These results provide support for Hypothesis 1a, that individuals expect lenient treatment for transgressing when it is their birthday, particularly if they mentioned it.

#### Study 1b

This study solicited individuals’ intuitions about they would do if they were stopped for drinking and driving on their birthday, as well as reasons behind their choice.

##### Participants and procedure

We paid 112 participants (56% male; *M*_age_ = 34 years, *SD* = 10.5) $0.50 to answer five questions and complete some basic demographic information. Participants read:

It is your birthday, and you’ve been out with friends celebrating. While driving home, you get pulled over by a police officer and asked to take a breathalyzer test. In your interactions with the driver, do you mention to the police officer that it is your birthday?

We then asked them to indicate (on a 5-point scale) to what extent they agreed with four statements about why they might have made the choice they did: (1) It would result in the most lenient treatment from the officer; (2) It was the best excuse for my behavior; (3) It was the most appropriate choice to make; and (4) It would be the easiest thing to do.

##### Results

We did not include an attention check in this study, so results are reported for the whole sample. Thirty-five of the respondents (31%) said that they would mention their birthday to the officer. Those who said they would mention their birthday to the officer reported significantly higher levels of agreement with the statements that doing so: (1) would result in more lenient treatment from the officer [*M*_mentioned_ = 3.31, *SD* = 1.02 vs. *M*_not mentioned_ = 2.05, *SD* = 0.83, *t*(110) = 6.94, *p* < 0.001], (2) was the best excuse for their behavior [*M*_mentioned_= 3.20, *SD* = 1.16 vs. *M*_not mentioned_= 1.83, *SD* = 0.79, *t*(110) = 7.33, *p* < 0.001], and (3) would be the easiest thing to do [*M*_mentioned_= 3.69, *SD* = 0.90 vs. *M*_not mentioned_= 2.78, *SD* = 1.19, *t*(110) = 4.02, *p* < 0.001]. Both groups reported their choice was equally appropriate [*M*_mentioned_= 3.26, *SD* = 0.78 vs. *M*_not mentioned_= 3.29, *SD* = 1.36, *t*(110) = 0.12, *p* = 0.91]. These results provide some support for Hypothesis 1b. A substantial minority of individuals claim that they would mention it was their birthday to a police officer if they transgressed on their birthday. In addition, consistent with Hypothesis 1a, individuals who reported they would mention it was their birthday expected that doing so would lead to more lenient punishment for their offense.

#### Study 1c

In our final scenario study, we asked participants to imagine themselves in the role of the police officer. We wanted to see if the leniency they predicted they would receive as the driver would translate when they imagined themselves in the role of the police officer. We also wanted to assess how individuals in the role of the authority reacted to different ways that drivers might make their birthday salient, as a preliminary test of Hypothesis 2.

##### Participants and procedure

We paid 273 participants (62% male; *M*_age_= 32 years, *SD* = 9.8) $0.50 to respond to a scenario. The experiment had four conditions: a control condition, and three birthday conditions (mentioned, soliciting-leniency, and noticed). We included several different birthday conditions to develop a more complete understanding of the outcomes of a range of possible interactions between the driver and officer. The scenario read:

Imagine you are a police officer conducting a road patrol. When you stop the next driver, you notice a faint smell of alcohol, though he is speaking clearly. To be safe, you require him to take a breathalyzer test. It turns out his blood alcohol content is 0.075%. The driver is under the 0.08% legal limit for Blood Alcohol Content (BAC), so you are not required to arrest him. However, you’re concerned the breathalyzer test might not accurately reflect the impairment level of the driver, so you might want to arrest him as well.

[**Control**] *As you consider your decision, he tells you that he is on his way home from dinner.*

[**Birthday-mentioned**] *As you consider your decision, he tells you that he is on his way home from dinner, and mentions that it is his birthday today.*

[**Birthday-soliciting-leniency**] *As you consider your decision, he tells you that he is on his way home from dinner, and mentions that since it is his birthday today, it would be nice for you to let him go with a warning.*

[**Birthday-noticed**] *As you consider your decision, he tells you that he is on his way home from dinner. As you take his driver’s license back to your vehicle for some paperwork, you happen to notice that today is the driver’s birthday.*

Participants were then asked to make a forced choice prediction about whether they would arrest the driver or release them with a warning.

We also tested individuals’ psychological reactions to the scenarios. We used a 3-item measure of threat to freedom that has been used to study psychological reactance (Dillard and Shen, 2005). The items (“The driver tried to make my decision for me,” “The driver was trying to manipulate me,” and “The driver was trying to pressure me”) were measured on a 5-point scale from strongly disagree to strongly agree (α = 0.91). In addition, reactance theory suggested that threats to one’s perceived autonomy might trigger “hostile and aggressive feelings” (Brehm, 1966, p. 9), though the theory claims that reactance may be present regardless of whether it is accompanied by such emotions. Dillard and Shen (2005) measured this type of negative affect using four items (irritated, angry, annoyed, aggravated), on a 5-point scale that ranged from “not at all” to “to a large extent” (α = 0.92).

##### Results

Four participants failed the attention check question in this study; results are reported for the remaining 269 participants. There were significant differences in whether participants reported they would arrest the driver, χ^2^(3, *N* = 269) = 12.95, *p* = 0.005. In the control condition, 21% said they would arrest the driver, which was not significantly different from the 16% who said they would arrest the driver in the birthday-mentioned condition, χ^2^(1, *N* = 135) = 39, *p* = 0.53, nor the 12% who said they would arrest the driver in the birthday-noticed condition, χ^2^(1, *N* = 135) = 1.85, *p* = 0.17. However, when the driver mentioned it was his birthday in an effort to solicit leniency, individuals were significantly more likely (36%) to predict they would arrest the driver, χ^2^(1, *N* = 135) = 3.87, *p* = 0.049 than in the control condition.

The scenarios also elicited different levels of psychological reactance in the participants, *F*(3,265) = 35.41, *p* < 0.001. Results showed a significant linear trend, *F*(1,265) = 83.56, *p* < 0.001, such that participants were significantly more likely to perceive a threat to their freedom as the driver made the birthday increasingly salient. The least reactance was reported in the control condition (*M* = 1.95, *SD* = 0.93) and the birthday noticed condition (*M* = 1.74, *SD* = 0.93), which did not differ from each other (*p* = 0.22). This level rose significantly in the birthday-mentioned condition (*M* = 2.68, *SD* = 0.93, *p* < 0.001) and again in the birthday-soliciting-leniency condition (*M* = 3.30, *SD* = 1.07, *p* < 0.001). The difference between the birthday-mentioned and birthday-soliciting-leniency conditions was also significant (*p* < 0.001).

Participants’ negative affect also significantly differed by condition, *F*(3,265) = 5.44, *p* < 0.001. However, the birthday-soliciting-leniency condition (*M* = 2.24, *SD* = 1.06) was the only condition that significantly differed from the rest (all at *p* < 0.001), which were statistically indistinguishable from each other (*M*_birthday-mentioned_= 1.70, *SD* = 0.86; *M*_birthday-noticed_= 1.63, *SD* = 0.83; *M*_control_ = 1.69, *SD* = 0.81).

These results provide preliminary support for Hypothesis 2, that a transgressor’s birthday elicits negative psychological reactions among individuals with discretion over their punishment. In addition, the more obvious the effort to capitalize on the social expectation of leniency, the more negative the reaction.

#### Discussion

Together, these results suggest three things. First, birthdays do represent a social context in which individuals expect to receive lenient treatment for their transgressions – even if the transgression is quite severe. Second, though still a minority, a substantial proportion of individuals claim they would mention it was their birthday to a police officer if they were pulled over for drunk driving on that day. Third, individuals imagining themselves in the role of a police officer believe they would only treat birthday offenders more stringently if the driver attempted to use that fact to solicit lenient treatment for his offense. Third, even though respondents reported they would only treat birthday offenders more stringently if they used the birthday to solicit leniency (this was also the only condition that elicited significantly more negative affect from the respondent), any mention of the transgressor’s birthday elicited psychological reactance. This last finding suggests that individuals with discretion over punishment may have more general psychological reactions to birthday transgressors.

### Study 2: Field Evidence from Drunk Driving Stops by Officers

In Study 2, we use a unique sample of field data to identify how individuals in positions of authority actually penalize transgressions in a social context that elicits expectations of leniency. Specifically, we study arrests involving suspicion of DUI of alcohol in the state of Washington, and test whether otherwise similar drivers are more likely to be arrested if it is their birthday, compared to those for whom it is not their birthday.

#### Empirical Context

In all U.S. states, driving while intoxicated by alcohol (drunk driving) is prohibited and has a severe impact on public safety. Economists have estimated that intoxicated drivers create externalities of at least 30 cents per mile driven due to social welfare costs of traffic fatalities (Levitt and Porter, 2001). Alcohol-related fatalities in the United States were estimated to be 11,948 in 2010, representing 36% of all traffic fatalities that year (National Highway Traffic Safety Administration, 2010). Furthermore, deterring drunk driving is difficult, with estimates that only one out of every 2,000 drunk drivers is actually arrested (personal communication, Washington State Patrol).

**FIGURE 1:**
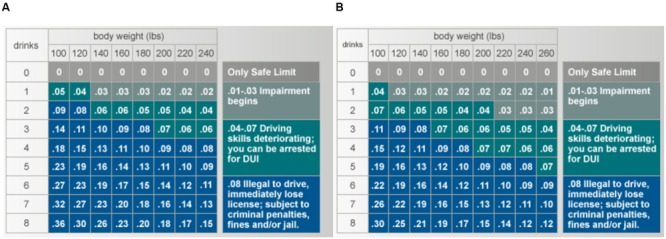
**Relationship between Alcohol Consumption and BAC. (A)** Represents the average blood alcohol content for women based on number of drinks (12 oz. beer, 5 oz. wine, 1.5 oz. hard alcohol) and body weight. **(B)** Represents men.

Driving under the influence laws are enforced by several police agencies in Washington State, including the Washington State Patrol, which is responsible for monitoring and enforcing the state’s highway systems, as well as local agencies, including municipal police, county sheriff’s offices, and Indian Nations agencies. In Washington State, DUI laws are primarily based on the driver’s blood alcohol level (BAC). Drivers whose BAC exceeds 0.08% are said to be in *per se* violation of state law and have little legal defense. Such drivers face minimum penalties of $865, 24 h incarceration, and 90 days suspended license for their first offense. Drivers with BAC levels above 0.15% are subject to even greater penalties, including minimum fines of $1,120, 2 days incarceration, and a 1-year revocation of one’s driver’s license. Penalties escalate rapidly with repeat offenses. **Figure 1** presents the average relationship between drinking behavior and BAC, conditional on gender and body weight, though food consumption, regular alcohol consumption, and genetic factors also influence BAC. As the body processes alcohol, BAC drops at an average rate of 0.015 per hour.

When an officer suspects a driver of DUI, she typically administers a field sobriety test. Furthermore, the officer administers a mobile breath test (“breathalyzer”), which estimates the BAC of the driver. If the officer determines the driver to be intoxicated, the driver is placed under arrest and taken to a field station for a formal (and admissible in court) breath test. If the officer observes a mobile BAC greater than 0.08, the decision is straightforward. The driver is almost certainly in *per se* violation, and the officer arrests the driver. However, the decision is much less clear if the mobile BAC is below 0.08. When the mobile BAC is below 0.08, the officer has discretion over whether or not to arrest the driver. Drivers with BAC levels between 0.04 and 0.079, for example, are likely impaired, but less so than *per se* violators. These “marginal offenders” are arrested at the discretion of the officer. We use the term “marginal” to refer to drivers who fall just below the *per se* blood alcohol threshold^3^.

Arresting the driver presents several potential costs for the officer. First, the arrest process takes the officer off the road for several hours and thereby precludes her from potentially arresting an even more highly intoxicated driver. Second, drivers who do not violate the *per se* rule are much more difficult to prosecute, as a conviction must rely on the officer’s evaluation of the driver’s intoxication. Consequently, prosecuting attorneys typically discourage officers from arresting drivers with low BAC, and most of these cases are plea-bargained (decided without going to court) with minimal penalties.

#### Data

Our data include every DUI arrest in Washington State from 2001 to 2009. These data include the agency and identity of the arresting officer as well as the name, age, gender, and ethnicity of the driver. Also included are the date, time, and location of the arrest. The data also note the primary criminal charge, which allows us to exclude DUI arrests that are secondary to more severe crimes such as weapons violations, violent crimes, or outstanding arrest warrants. The data also identify the mobile BAC reading, when taken, as well as the court-admissible BAC reading from the police station. Since the data also identify the exact time of each test, we know the length of delay before the driver was given the court-admissible test. We present basic summary statistics in **Table 1** for both the pooled sample as well as the sample separated by birthday/non-birthday. The average BAC for all arrests is 0.13, with 94% above the *per se* threshold. Approximately one out of every 300 arrests is a birthday driver. The average age of the sample is 34, 21% are female and 81% are ethnically white (non-Hispanic).

**Table 1:** Study 2: Descriptive statistics for DUI arrests.

	All arrests		Birthday arrests	Other arrests
Variable	Mean	*SD*	Mean	Mean
Field BAC	0.133	0.049	0.134	0.134
*Per se* violation	0.94	0.24	0.92	0.94
Field BAC – Station BAC	-2.18	35.12	-1.06	-2.18
Minutes from field to station	60.73	46.75	59.17	60.74
Birthday driver	0.004	0.062	1	0
Driver age	34.33	11.38	37.08	34.32
Female driver	0.21	0.41	0.23	0.21
White driver	0.83	0.38	0.82	0.83
Number of observations	134,507		518	133,989

One weakness in our data is that we are unable to observe drivers stopped for suspicion of DUI but not arrested. Only drivers who were arrested appear in our data, creating potential survivor bias in any standard regression analysis. We will address this weakness by exploiting the discrete threshold at BAC = 0.08 in order to infer distributions of non-arrested drivers in the data. Another weakness is the relative rarity of birthdays, which represent 0.38% of all arrests vs. 0.27% (one out of 365.25) of all days. This rarity means that we must infer differences in birthday traffic stops from a substantially smaller sample than the total DUI database.

#### Identification Strategy

Using driver’s birthday as the context in which there is a social expectation of lenient treatment has several important characteristics from an identification perspective. First, the norm of preferential treatment on one’s birthday is universally known and widely observed. Second, birthdays are unobservable to an officer prior to a traffic stop and thus unlikely to create an unobservable selection bias in traffic stops. Third, birthdays are randomly distributed and uncorrelated with other factors that might affect officer leniency. This third point is critical for our decision to examine birthdays instead of other holidays such as Valentine’s Day, Mother’s Day, or Christmas, which may affect the officer. An officer showing leniency on Valentine’s Day, for example, may simply want to avoid a 2 h arrest that keeps him from dinner with a spouse, or he may be in a foul mood due to working on a holiday.

We identify officer stringency in DUI enforcement by observing how often officers arrest *per se* offenders relative to marginal offenders. While all officers must arrest *per se* offenders, extremely stringent enforcement would entail an increase in arrested marginal offenders relative to *per se* arrests. Officers may be able to identify extremely intoxicated drivers (e.g., BAC > 0.15) before a traffic stop, but it is unlikely they would be able to *ex ante* distinguish between marginal offenders and those with BAC levels just above the *per se* limit. Consequently, the ratio of traffic stops that involve BACs just above the threshold (e.g., BAC = 0.08) should be approximately equal to the frequency involving BACs just below (e.g., BAC = 0.079), as should the appearance of intoxication when the driver is first confronted. Given the approximately equal number of marginal and *per se* violators stopped and tested, the relative frequency of arrest of marginal offenders relative to borderline *per se* offenders is unlikely to reflect the choice to stop drivers and instead will reflect the decision to punish marginal offenders. This approach is similar to one recently used to examine possible racial bias in DUI stops (Horn et al., 2014).

#### Results

**FIGURE 2:**
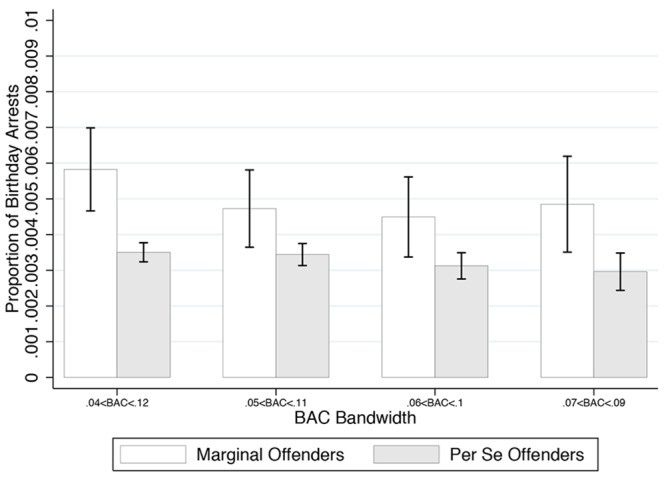
**Study 2: Proportion of arrests of birthday drivers for different bandwidths near the *per se* threshold.** Marginal offenders represent those drivers with BAC below the 0.08 threshold. *Per se* offenders are above the threshold. Bars represent ±1 SE.

We first present birthday arrest frequency for marginal and *per se* violators for four different bandwidths surrounding the 0.08 *per se* threshold (see **Figure 2**). The white bar represents marginal offenders, while the gray bar reflects *per se* violators. Whiskers reflect plus or minus one standard error. The four decreasing bandwidths are represented from left to right, with the furthest left group indicating plus or minus 0.04 and the furthest right group representing plus or minus 0.01. **Figure 2** shows a much higher level of birthday arrests for marginal offenders than *per se* offenders, which suggests that discretion leads to increased stringency for birthday drivers stopped by police. Together, these results provide support for Hypothesis 3a, that birthday drivers receive increased stringency rather than increased leniency.

##### Regression analysis

The goal of our analysis is to identify how the behavioral interaction between the transgressor (driver) and punisher (officer) are affected by the expectation of leniency associated with birthdays. Consequently, we are concerned that the increased likelihood of a discretionary arrest on birthdays might simply reflect fundamental differences between the characteristics of birthday and non-birthday drivers. Similarly, the police who arrest them or the conditions under which they are arrested might be different. To address this, we implement regression analysis that estimates a decrease in the likelihood of a birthday driver for all arrests above the *per se* threshold. This approach is similar to a regression discontinuity design, which involves estimating the impact of a discrete threshold in a continuous independent variable on an outcome variable (Imbens and Lemieux, 2008; Snyder, 2010; Pierce and Snyder, 2012; Pierce et al., 2013). We cannot achieve the standards of a true regression discontinuity design, because the extremely rare occurrence of birthday arrests does not provide sufficient observations very close (i.e., BAC values 0.079 and 0.08) to the *per se* threshold. We therefore urge caution in interpreting any causal relationship from our data.

Because officers cannot observe on which side of the threshold a moderately intoxicated driver lies before arrest, the assignment near the threshold is random for those stopped for DUI. Since DUI stops are randomly assigned to either side of the threshold, our theoretical argument is that the behavioral interaction between the driver and officer is the mechanism driving any discrete increase in birthday probability at the *per se* threshold. Since this mechanism is difficult to directly identify in the arrest data, the purpose of our regression model is to provide evidence that this difference is not due to observable driver, officer, or arrest characteristics that are correlated with birthdays but different from our argued mechanisms.

Our first specification uses logistic regression to estimate the probability that an arrest involves a driver birthday as a function of the *per se* rule. If officers treated birthday drivers identically to other drivers, we should expect arrests immediately on each side of the threshold to have equal probability of involving a birthday. Alternatively, if officers are more aggressive in punishing marginally drunk drivers on their birthday, we should expect a higher probability of a birthday for BAC < 0.08 and therefore a negative coefficient for the *per se* threshold. It is important to account for the underlying relationship between the dependent variable (birthday) and the continuous variable that defines the discrete threshold (BAC). We include a quartic polynomial of BAC as a control variable to allow for functional flexibility in the relationship between drinking behavior and birthdays.

Our base model with no control variables is presented in column 1 of **Table 2**, with logit coefficients and robust standard errors clustered at the officer level in parentheses. Column 2 adds flexible time controls, and column 3 adds controls for driver age (quartic polynomial), gender, and ethnicity. Also included are dummy variables for each county. Each column shows a negative relationship between the *per se* threshold and the probability of an arrestee birthday. The correct interpretation for these results is that the probability of an arrestee birthday distinctly drops when the BAC level crosses the threshold at 0.08% (thus removing the officer’s discretion). Providing support for Hypothesis 3a, these models suggest that marginally drunk drivers who are stopped are more likely to be arrested on their birthday than on other days. To aid interpretation, we calculate the marginal effects for the fully controlled model, which are 0.0018 (*p* = 0.06). Given the base rate of birthday arrestees of approximately 0.4% of *per se* violators, one’s probability of arrest increases by about 50% near the 0.08 BAC threshold on one’s birthday.

**Table 2:** Study 2: Regression models predicting birthday arrests.

	(1)	(2)	(3)	(4)
	Logit	Logit	Logit	OLS
Driver sample:	All	All	All	All
Dependent variable:	Birthday	Birthday	Birthday	Birthday
*Per se* violator	-0.543^∗^ (0.265)	-0.536^∗^ (0.265)	-0.488^†^ (0.259)	-0.0021 (0.0013)
BAC	0.008 (0.013)	0.009 (0.013)	0.006 (0.013)	0.00003 (0.00005)
BAC^2^	-0.00003 (0.0001)	-0.00004 (0.0001)	-6.3*e*-06 (1.3*e*-04)	-3.1*e*-08 (5.5*e*-07)
BAC^3^	5.3*e*-08 (5.4*e*-07)	7.5*e*-08 (5.5*e*-07)	-3.8*e*-08 (5.4*e*-07)	-4.7*e*-10 (2.1*e*-09)
BAC^4^	-8.4*e*-11 (7.4*e*-10)	-1.1*e*-10 (7.5*e*-10)	2.8*e*-11 (7.3*e*-10)	8.4*e*-3 (2.7*e*-12)
Month/Day dummies	No	Yes	Yes	Yes
Year dummies	No	Yes	Yes	Yes
Age	No	No	-0.363^†^ (0.217)	-0.028^∗∗^ (0.003)
Age^2^	No	No	0.012^†^ (0.006)	0.001^∗∗^ (0.0001)
Age^3^	No	No	-0.0002^†^ (0.0001)	-0.00002^∗∗^ (1.7*e*-06)
Age^4^	No	No	9.1*e*-07^∗^ (4.1*e*-07)	9.4*e*-08^∗∗^ (9.3*e*-09)
Male	No	No	-0.060 (0.112)	-0.00007 (0.0005)
Driver ethnicity dummies	No	No	Yes	Yes
County dummies	No	No	Yes	Yes
Officer FE	No	No	No	Yes
Pseudo R-squared	0.0007	0.0370	0.0412	0.0451
Number of observations	134,507	134,507	133,795	134,507

*Standard errors clustered at the officer level in parentheses. ^†^*p* < 0.10; ^∗^*p* < 0.05; ^∗∗^*p* < 0.01. Observations across models are not equal due to maximum likelihood estimation dropping perfectly predicted groups. Fixed effects in Model 4 are at the officer level. *p*-value for Model 4 is 0.11. Small coefficients and standard errors listed in scientific notation.*

As a robustness test, in column 4 we report a linear probability model with officer fixed effects, since the rarity of birthday events does not allow the use of logit models with fixed effects. The linear fixed effect model produces a coefficient very similar to our marginal effects, but with reduced statistical significance (*p* = 0.11), which is unsurprising given the coefficient is only identified off the smaller set of officers with at least one birthday arrest. Still, this model suggests that our results cannot be explained by the most stringent officers stopping birthday drivers. Other multi-level models that might explore agency- or officer-level predictors of stringency cannot be estimated, because few officers or agencies experience more than one or two birthday arrests.

We note that we cannot estimate the marginal effect at other points farther below the threshold due to our identification strategy. Furthermore, the low number of birthday arrests in our data makes more formal testing of regression discontinuity models difficult, so our results would be more compelling if we could triangulate them using additional data.

To address possible differences in our non-birthday and birthday samples, we also created a matched sample based on observable driver demographics, BAC, and stop characteristics. We implement a propensity score matching algorithm that chooses the ten nearest non-birthday neighbors for each birthday arrest, which reduces our sample to 5,117 arrests (some non-birthday arrests are neighbors for multiple birthday arrests). Using this matched sample, we repeat the *t*-tests and regressions reported in **Figure 2** and **Table 2**; these produce nearly equivalent results. The proportion of birthday offenders remains higher for marginal offenders for each of the four bandwidths used in **Figure 2** (*p* < 0.01), and the birthday coefficients for the three models presented in **Table 2** are very similar, despite a 96% decrease in sample size: -0.523 (*p* = 0.06), -0.496 (*p* = 0.08), -0.482 (*p* = 0.09).

##### Robustness tests

Our evidence of higher stringency toward birthday drivers supports Hypothesis 3a, but raises a number of alternative explanations. One natural concern with our identification strategy is that BAC readings may not accurately represent the public safety risks of birthday drivers, and that the increased stringency we observe represents a rational police response to expectations of future accidents or increasing intoxication. We systematically examine these alternative explanations by testing differences in arrested drivers’ characteristics.

We first address whether the mobile BAC of birthday drivers stopped by police accurately reflects their level of alcohol consumption, relative to other drivers. In this alternative explanation, marginally drunk birthday drivers have alcohol in their stomach that has not yet entered the bloodstream due to binge drinking or a stomach full of food, and officers arrest the driver because of their tacit knowledge that their BAC will continue to climb above the *per se* threshold later in the evening. In such a case, the decision to disproportionately arrest marginally drunk birthday drivers would be rational and show great foresight. To examine whether birthday drivers are more likely to increase in BAC due to pre-stop drinking patterns, we examine the change in BAC between the mobile and station tests. This change reflects how much the driver’s BAC increased or decreased between arrest and arrival at the testing facility during a time where additional drinking was not possible. The differences between average BAC changes of birthday (-0.0011) and non-birthday (-0.0022) drivers are indistinguishable (*p* = 0.50), as are the number of minutes between the two tests (60.4 vs. 59.1, *p* = 0.51). This suggests that BAC measures reflect equal intoxication of birthday and other drivers.

We next address the alternative explanation that marginally drunk birthday drivers may be inherently more dangerous than their non-birthday counterparts and that stringency toward them is a rational public safety response. For this alternative explanation, we test whether birthday drivers are more likely to drink (and drive) later in the evening, making their arrest a pre-emptive strategy for law enforcement. The average time of arrest is also nearly identical between the two groups (10:54 p.m. vs. 10:59 p.m., *p* = 0.58), suggesting that officers are not preemptively arresting birthday drivers earlier in the evening to avoid later drinking and driving.

We also examined whether arrested birthday drivers were more likely to have it be their first DUI arrest (in the state) compared to other drivers. To do so, we used only those drivers where no officer discretion was involved (thus eliminating any birthday bias), and found that although it was somewhat more likely that birthday drivers were being arrested for the first time compared to non-birthday drivers (91% vs. 89%), the difference was statistically indistinguishable (Fisher’s exact test, *p* = 0.19).

Another alternative explanation is that officers might punish marginally drunk birthday drivers more stringently because they believe birthday drivers are more likely to be “scared straight” by the arrest. Although we cannot observe other confounds (such as differences in conviction and sentencing), we tested whether those whose first arrest was on their birthday were less likely to be arrested for a later DUI. For *per se* violators (where discretion was not involved), birthday drivers were slightly less likely to reoffend (17% vs. 19%, Fisher’s exact test, *p* = 0.11), but there is virtually no difference among the lowest *per se* offenders who best approximate marginal offenders (BAC between 0.08 and 0.12). Birthday and other drivers both reoffended at a 17% rate (*p* = 0.99). These suggest that there is no strong deterrence reason why officers should arrest birthday offenders more often than other offenders. Even if they are, it is not effective. Marginal birthday offenders are, if anything, more likely than others to reoffend after being arrested (30% vs. 17%, Fisher’s exact test, *p* = 0.10).

Finally, we examine whether intoxicated birthday drivers were more likely to be involved in an accident compared to other drunk drivers. Arresting after an accident where the driver has a positive BAC involve no police discretion (hence we had excluded arrests involving accidents from our main sample). To test whether officers may be arresting birthday drivers at a higher rate because they have insider knowledge that they are more likely than other drivers to cause later accidents, we compare the ratio of birthday drivers among those arrested for DUI offenses ending in accidents (arrests excluded from our main sample) to the ratio of birthday drivers among discretionary DUI arrests. The percentage of drunk-driving accidents involving birthday drivers is 0.43%, compared to 0.41% for discretionary officer arrests (*p* = 0.39), suggesting that birthday drivers are no more likely to get into DUI accidents than drivers on other days.

This similarity in birthday rates for non-discretionary accident rates also casts doubt on an alternative explanation that officers give fewer breath tests to birthday drivers, and consequently might show more stringency toward those under 0.08 to compensate for this prior leniency. If that were the case, then we would expect a lower average rate of birthday drivers in discretionary tests (non-accidents) than in mandatory ones, which we do not. Together, these tests cast doubt on alternative explanations for police stringency toward birthday drivers, but of course cannot disprove them.

#### Discussion

Of course, we cannot know whether drivers are actually soliciting leniency in their interactions with police officers when they are pulled over on their birthdays. However, if Study 1b is any indication, a substantial minority of the individuals (31% of the study sample) reported that they would mention their birthday to the police officer. Alternatively, contrary to Study 1c, which suggested that reactance in the condition in which participants noticed the birthday was equal to the control condition, officers in the field may react negatively to drunk driver even if their birthday isn’t mentioned. Whatever occurs between the officers and the drivers in the field, our analysis provides support for Hypothesis 3a: drivers who are at the margins of the legal limit for blood alcohol are more likely to be arrested on their birthday than on other days. This effect appears to be unrelated to the public safety risk of these drivers, their demographics, and the conditions under which they are arrested.

### Study 3: Testing Psychological Reactance as a Mechanism in the Lab

The data from Study 2 do not allow us to test whether psychological reactance explains the apparent stringency toward birthday drivers, neither can they reveal whether this is a more general behavioral response or whether it is idiosyncratic to the setting of drunk driving. We address these concerns by designing a laboratory experiment using a different type of transgression, which additionally allows us to test psychological reactance as our hypothesized mechanism (Hypothesis 3b).

#### Participants and Procedure

The behavioral lab at a UK-based business school (43% male; *M*_age_ = 28.5 years, *SD* = 9.7) recruited 162 participants to complete the study for a £10 payment. The study was approved by the school’s Ethics Review Board, and met all APA requirements for the ethical treatment of research participants.

Participants were randomly assigned to one of three conditions. There were two birthday conditions: one in which the transgressor was using his birthday as a reason to solicit preferential treatment (**birthday-soliciting-leniency**), and one in which the transgressor merely mentioned it was his or her birthday (**birthday-mentioned**). A control condition made no birthday reference.

We informed participants that the lab was partnering with a nearby school specializing in English as a Second Language to evaluate a student essay-writing competition. Participants were all assigned to the role of “evaluator” and tasked to judge three of the essays competing for prizes. We also told participants that, because teachers typically know the students in their classes before grading any of their work, the students had written a short paragraph about themselves, which would be attached to each essay. We used actual example essays from the American College Testing writing assessment arguing in favor of extending high school by 1 year. We chose two essays that the assessment service used as examples of poorly written essays (that had scored 1 and 2 out of 5) and one example of a good essay (that had scored 5 out of 5).

We provided participants with a scoring sheet and contest rules, which included a rule forbidding essays over 500 words from being eligible for prizes. The rule read**: “*The students were instructed to follow a 500-word limit. You should still grade their essay if it is more than 500 words, but if they exceed 500 words, it is ineligible for the prize.*”** They were instructed to judge each essay and asked whether they nominated any of the essays for either the first prize (a 10% tuition fee refund), or an honorable mention (a new backpack with the school logo). Finally, they were told that the students were aware that the essays were being evaluated by outside graders and that competition winners, chosen by them, would be announced “this coming Friday.” Instructions stressed that the competition had meaningful outcomes for the students and that it was important for them to take their job seriously. Each participant evaluated the same three essays; however, the handwritten personal statements stapled to the essays varied. There were three versions: one written by a Brazilian female, one by a Mexican male, and one by a Spaniard whose gender was not made explicit. Personal statements were counterbalanced to ensure that any differences in participants’ evaluations or prize nominations were unrelated to the personal messages’ content, other than the birthday manipulation.

The birthday manipulation was included at the end of the personal statement attached to Essay #3 (the essay assessed by the American College Testing service as the one of the highest quality), which was always positioned last in the package. The handwritten personal statement also included a message that either mentioned the essay-writer’s birthday [**birthday-mentioned**: “*It’s my birthday next Friday, and I will be 22 years!*”], or suggested that the essay-writer deserved the prize because it was their birthday [**birthday-soliciting-leniency**: *“I really think I deserve the prize because it will be my birthday the day the prizes are announced—I will be 22 years!”*]. In the control condition, nothing was mentioned about the essay-writer’s birthday. This manipulation allows us to test whether the participants who appeared to solicit leniency because it was their birthday would be penalized more harshly, or whether simply mentioning the birthday would be enough to elicit the stringency effect we observed in the drunk driving data.

#### Measures

Participants were instructed to grade the essay’s unique ideas (10 points), persuasiveness (10 points), language quality (10 points), and grammar, spelling and punctuation (10 points). These points were summed to create a total score. We used participants’ scores as a manipulation check to confirm that the essay containing the birthday manipulation was evaluated as the “best” essay among the three, and thus the most likely to be nominated for a prize *if* the essay writer was not penalized for breaking the word limit rule.

##### Mechanism: psychological reactance

We measured participants’ psychological reactance to each of the essay writers’ personal statements using the same 3-item measure of threat to freedom used in Study 1c (Dillard and Shen, 2005). We measured the items for each essay writer (α = 0.83 for essay writer 1, α = 0.86 for essay writer 2, and α = 0.82 for essay writer 3). To rule out the alternative explanation that negative affect (anger or annoyance) was driving our effects, we also included the same measure of negative affect used in Study 1c (α = 0.82 for essay writer 1, α = 0.86 for essay writer 2, and α = 0.82 for essay writer 3).

##### Dependent variable: stringency

The word counts of each essay were handwritten on each of the essays and circled. At 513 words, Essay #3 violated the 500-word limit rule by 13 words. Neither of the other two essays violated the word limit. The dependent variable of interest was whether participants treated Essay #3 with increased stringency by not nominating it for the prize even though it was the best of the three essays.

#### Results

Five participants failed to complete all relevant measures, and were excluded from the analysis. Results are reported for the remaining 157 participants.

A repeated measures ANOVA with final score as the within-subjects factor confirmed the ranking of the essays provided by the American College Testing service. The essay scores significantly differed from each other, *F*(2,155) = 326.55, *p* < 0.001, and the score for Essay #3 (*M* = 32.0, *SD* = 5.9) was significantly higher than the scores for Essay #1 (*M* = 23.1, *SD* = 5.6) and Essay #2 (*M* = 15.4, *SD* = 6.6) scores. Thus, we interpret the failure to nominate Essay #3 for the prize as evidence that evaluators were penalizing this student for transgressing the word limit rule, effectively disqualifying the writer from the competition, rather than as evidence that the evaluator believed the essay to be low quality.

We next established that participants’ psychological reactance to the third essay was affected by the condition to which they were assigned, *F*(2,154) = 3.98, *p* = 0.021. Consistent with Study 1c, this pattern followed a significant linear trend, *F*(1,154) = 7.95, *p* = 0.005. The highest levels of psychological reactance were felt by participants in the birthday-soliciting-leniency condition (*M* = 2.83, *SD* = 1.14), with slightly lower levels by participants in the birthday-mentioned condition (*M* = 2.58, *SD* = 0.91), and lowest levels in the control condition (*M* = 2.27, *SD* = 0.96). Participants clearly reacted more strongly as messages reflected more explicit attempts to capitalize on expectations that they would be treated preferentially on their birthday. Participants’ levels of negative affective reaction to the third essay did not differ by condition, *F*(2,154) = 0.11, *p* = 0.89, *M*_birthday-mentioned_ = 1.21, *SD* = 0.47; *M*_birthday-soliciting-leniency_ = 1.26, *SD* = 0.53; *M*_control_ = 1.25, *SD* = 0.58. However, we note that our threat to freedom measure correlates with our measure of negative affect (*r* = 0.44, *p* < 0.001), indicating that a threat to freedom is experienced, in part, as negative affect.

Hypothesis 3b predicted that an authority figure’s increased stringency (in this case, penalizing those who violated competition rules) as a function of targets’ birthdays is driven by psychological reactance. We used Preacher and Hayes’ PROCESS macro (Hayes, 2013) to test psychological reactance as the mediator in the relationship between transgressors’ birthday statuses and whether they were denied the prize. Our design uses a dichotomous outcome variable and a multi-categorical independent variable. To test our predicted relationships, we constructed dummy variables for each condition (birthday-soliciting-leniency, birthday-mentioned, and control). For each model, one dummy variable is specified as the independent variable and a second dummy variable is included as a covariate; the resulting test of the indirect effect represents the comparison between the condition specified as the independent variable and the reference condition (excluded from the analysis). The macro generates bias-corrected bootstrap confidence intervals for each indirect effect.

We ran three models for all the relevant comparisons, each using 5,000 bootstrap samples. We included the participants’ reactance toward the first and second essays as covariates in the analyses, as the reactance measures were significantly correlated with each other (between Essay 1 and Essay 2, *r* = 0.68, *p* < 0.001, between Essay 1 and Essay 3, *r* = 0.37, *p* < 0.001; and between Essay 2 and Essay 3, *r* = 0.39, *p* < 0.001), and we wanted to ensure that our models used the reactance triggered by our birthday manipulation as the mediator of our effects, rather than the reactance the essay writers’ messages elicited overall. Results for all three models are reported in **Table 3**. Compared to the control condition, the indirect effect of either birthday condition on increased stringency via psychological reactance was positive, with 95% confidence intervals that excluded zero, indicating significant indirect effects via reactance. The indirect effect was largest comparing the birthday-soliciting-leniency condition to the control condition (point estimate = 0.31, 95% CI 0.035 to 0.709). The 95% confidence internal for the birthday-mentioned condition compared to the control condition also excluded zero (point estimate = 0.15, 95% CI 0.011 to 0.424). The birthday condition in which the student explicitly solicited leniency also showed a bigger indirect effect compared to the birthday-mentioned condition (point estimate = 0.16, 95% CI 0.004 to 0.498). We ran this same set of models, including negative affect as the mediator rather than reactance, and in each case the indirect effect straddled zero, indicating that negative affect does not explain the increased stringency toward birthday offenders.

**Table 3:** Study 3: Model summary information comparing indirect effects of birthday and control conditions on stringency via psychological reactance.

	Consequent					
	M (psychological reactance)			Y (stringency in punishment)		
Antecedent	Coefficient	*SE*	*p*	Coefficient	*SE*	*p*
*For all models*						
M (Reactance to Essay #3)				0.42	0.19	0.028
Reactance to Essay #1	0.28	0.14	0.044	-0.68	0.34	0.048
Reactance to Essay #2	0.39	0.12	0.002	0.19	0.30	0.519
*Comparing Birthday-Soliciting-Leniency to Control*						
X (Birthday-Soliciting-Leniency)	0.72	0.18	<0.001	-0.11	0.45	0.813
AB (Effect of X on Y via M)				0.31	0.17	95% CI: 0.035 to 0.708
*Comparing Birthday-Mentioned to Control*						
X (Birthday-Mentioned)	0.34	0.18	0.057	0.09	0.42	0.835
AB (Effect of X on Y via M)				0.15	0.10	95% CI: 0.010 to 0.424
*Comparing Birthday-Soliciting-Leniency to Birthday-Mentioned*						
X (Birthday-Soliciting-Leniency)	0.38	0.18	0.034	-0.19	0.42	0.646
AB (Effect of X on Y via M)				0.16	0.12	95% CI: 0.004 to 0.498

*N = 157 for all models. Bias-corrected confidence intervals for the indirect (AB) effects based on 5,000 bootstrap samples.*

#### Discussion

In a substantially different paradigm and using a different type of transgression, Study 3 shows that psychological reactance to transgressors on their birthdays drives authority figures’ increased stringency toward them. It is interesting to note the subtlety of the birthday manipulations in this study: even in the birthday-soliciting-leniency condition, the student did not use his birthday as an excuse for violating the rules of the essay-writing contest, but merely said they deserved the prize because it was their birthday. These subtle manipulations help strengthen our argument that merely making the social context of a birthday salient increases how stringently a transgressor will be treated by an authority figure with the discretion to do so. Additional tests confirmed that psychological reactance – the subjective perception that one’s freedom is threatened – functions as a mechanism behind this effect. In contrast, we did not find empirical support for negative affect as a mechanism in this study.

### Study 4: Experimental Evidence on Bias Salience as an Alternative Mechanism

A second alternative explanation that could drive our results is overcompensation for bias. When attention is drawn to factors that may bias an individual’s evaluation of a target, a typical response is to try to correct for that possibility by adjusting the judgment away from the direction of the bias (Martin, 1986; Schwarz and Bless, 1992; Wegener and Petty, 1997). Given the challenges in correctly estimating the size of a potential bias, people often overcorrect for it in practice, leading to disproportional responses in the opposite direction (Wegener and Petty, 1995, 1997). We conducted another experiment in the same lab, to test whether evaluators’ stringency could be explained by a concern that they might be making a biased decision when it was the transgressor’s birthday.

#### Participants and Procedure

The behavioral lab at a UK-based business school (31% male; *M*_age_ = 25.7 years, *SD* = 8.3) recruited 101 participants to complete the study for a £10 payment. The experiment used the same experimental paradigm as Study 3, but employed a 2 (birthday-mentioned vs. control) × 2 (bias-salient vs. control) between-subjects design. The study was approved by the school’s Ethics Review Board, and met all APA requirements for the ethical treatment of research participants.

This experiment used a manipulation which simply mentioned the essay-writer’s birthday without actively soliciting leniency [**birthday-mentioned**: “*It would be so great to hear that I won first prize next Wednesday, because it’s my birthday that day and I’m already going to be celebrating with my friends!”* vs control: *“It would be so great to hear that I won first prize next Wednesday!”*]. In the **bias-salient** condition, the participants read these additional instructions: “*The leaders at the school are concerned that bias plays a role in who the teachers normally nominate to win this tuition discount. Therefore, you, as a lab participant, are helping us to understand if this bias is occurring, and if so, how it might be affecting student outcomes. Please be aware that what we know about people can sometimes bias our assessments of them. Try to be as UNBIASED in your assessments as possible.”* If bias salience was driving our effects, we should observe an interaction between the birthday-mentioned and bias-salient conditions, such that participants were more stringent for birthday essay writers in the bias-salient condition, compared to those in the control condition.

#### Results

We ran a logistic regression with stringency as the dependent variable, and birthday condition, bias condition, and their interaction as independent variables. The coefficients for the bias salience condition (*B* = -0.087, expB = 0.92, *p* = 0.93) and the interaction of the two conditions (*B* = -1.23, expB = 0.27, *p* = 0.31) were not significant, indicating that making the possibility of biased evaluations more salient to the participants did not strongly affect whether they treated the writer of Essay #3 with increased stringency. However, the same logistic regression revealed a significant, negative coefficient for the birthday-soliciting-leniency condition (*B* = 1.705, expB = 5.50, *p* = 0.046), indicating—consistent with the findings of Study 3—that Essay Writer #3 was treated with increased stringency in the birthday condition. Together, these results are consistent with Study 3, and suggest that overcompensation for bias is not a supported alternative mechanism for our effects.

## General Discussion

Our evidence from the field and the lab was consistent with the predictions of our theory. When confronted with a social expectation of lenient treatment, individuals with the authority and discretion to punish them treat transgressors more stringently rather than more leniently. In our studies, we used the transgressor’s birthday as a social context that leads to an expectation of lenient treatment, as it has many attractive characteristics that allow us to test this phenomenon in the field. Increased stringency for birthday transgressors, which we identified both in the field and in the lab, runs counter to what individuals believe happens to transgressors on their birthdays. We find that this effect is driven by psychological reactance toward the transgressor. Moreover, psychological reactance increases as the salience of the transgressor’s birthday increases (as, we assume, is the perception that the target is using his birthday to actively solicit lenient treatment).

### Theoretical Implications

Our results contribute to several literatures. First, our research contributes to a broader literature on punishment from psychological perspectives (Treviño, 1992; Fragale et al., 2009). Though theory has offered frameworks to evaluate when and how to punish (Arvey and Jones, 1985; Butterfield et al., 1996), examined its consequences (Ball et al., 1994; Podsakoff et al., 2006), and looked at how aspects of organizational context shape punishment decisions (Beyer and Trice, 1984), we know less about how the social context of transgressions affects punishment decisions. Even the literature on just deserts, which focuses on motivations to punish, has only addressed aspects of the social context that directly speak to the harm the act has caused or justifiable mitigating circumstances for it (such as the difference between intentional and accidental actions). However, there are many aspects of our context that may affect motivations to punish and punishment decisions, with only tenuous relevance to the transgression. We know little about aspects of our social context that ought to be unrelated to punishment decisions affect those decisions nevertheless. Our research addresses this gap by showing how subtle contextual factors (it being the transgressor’s birthday) play an important role in the ultimate penalties authorities impose.

Second, this paper contributes to our knowledge of how individuals behave when a context elicits two different motivations with conflicting behavior prescriptions. The large body of work in both psychology (e.g., Cialdini et al., 1991) and economics (e.g., Fehr and Gächter, 2002) that examines the power of expectations on individual behavior has focused primarily on how a single social norm motivates behavior. Instead, our work examines how individuals respond to multiple expectations elicited by the social context and that motivate us in conflicting ways, and how these motivational conflicts they may influence behavior.

Third, these findings extend our understanding of psychological reactance among individuals with the discretion over penalizing transgressions. Most of the literature on reactance has focused on refusals to help others (Berkowitz, 1973), engage in more positive behaviors, such as healthier lifestyle choices (Dillard and Shen, 2005), or pursue goals (Chartrand et al., 2007). These findings show that reactance also drives behaviors in punishment contexts, and confirms again that even very subtle messages can elicit perceptions that one’s freedom is being threatened, driving our behavior in the opposite direction.

Ultimately, these findings help us understand how discretion is exercised in the field, thus deepening our knowledge of how discrimination operates. Work on discrimination has focused almost exclusively on demographic characteristics such as age, race, ethnicity, and gender (Paluck and Green, 2009). Our research shows that other, less obvious factors will also lead individuals to treat transgressions differentially. This suggests that we need to extend our vigilance about how discretion may undermine the efficiency of punishment. It also deepens our understanding about the challenges humans have in debiasing their judgments and behavior (Wegener and Petty, 1995, 1997), particularly when individuals with discretion over how someone is treated interact with that person in advance of imposing penalties on them.

### Practical Implications

Our research also has important practical implications, both for alleged transgressors as well as those with discretion over punishing them—from managers and teachers to judges and jury members. Transgressors need to be aware that their intuitions about avoiding punishment by making leniency norms salient may backfire, resulting in harsher penalties than if they refrained from making the norm salient. In other words, transgressors may benefit from avoiding any perception that they are trying to capitalize on contextual factors that would suggest more lenient treatment. On the other hand, authorities with the responsibility to punish should be aware that in the face of conflicting motivations, they may make decisions that undermine the fairness, and ultimately the effectiveness, of their sanctions.

Compared to others responsible for punishing transgressions, law enforcement officials may be particularly likely to react negatively to perceptions that offenders are soliciting lenient treatment. They are accustomed to excuses and pleas for leniency from those they penalizing, to the extent that such pleas can become tiresome and prompt cynicism (Van Maanen, 1974). Research on leniency in law enforcement suggests that officer have “pet peeves,” including many related to the demeanor of offenders, that may elicit reactive and more severe responses (Schafer and Mastrofski, 2005). Contrition and verbally accepting responsibility for one’s actions may elicit more lenient responses from law enforcement, while soliciting special treatment may trigger reactive responses (Schafer and Mastrofski, 2005). Indeed, recent work by van Prooijen and Kerpershoek (2011) suggests that individuals may inflict excess retribution when given discretion to punish criminals, but only when they feel their autonomy is threatened. Though our data do not allow us to observe what specifically happens in the dyadic interactions between drivers and officers in our field data, our scenario studies suggest that driver behavior, and subsequent officer reactions to those behaviors, are critical to outcomes.

Our research also has important practical implications for managers, who are commonly given broad discretion to punish employees through oral reprimands, work suspension, or, in extreme cases, termination (Beyer and Trice, 1984; Butterfield et al., 1996). In fact, punishment is a widely used managerial strategy for producing desired changes in employee behavior (Ball et al., 1994). Thus, it is important for managers to know that they are also vulnerable to the challenges associated with managing contradictory motivations that might influence their actions.

### Conclusion

Our findings suggest that when authority figures have discretion over punishment decisions, making an expectation that a transgressor will be treated leniently salient leads to a negative psychological reaction, leading individuals with the authority to punish to do so more harshly. This might lead to the conclusion that discretion is overrated or overused. Yet, we do not want to suggest that our findings provide an argument against discretion, only a fair warning about some of its additional problematic qualities. Many dysfunctional consequences result when discretion is unavailable, such as under mandatory punishment guidelines (e.g., “three strikes” laws). These consequences include higher rates of violence and murders among repeat offenders and against witnesses of repeat offenses (Marvell and Moody, 2001; Zimring et al., 2001). Thus, eliminating discretion is likely not the answer. The message we take from our findings is that authorities with discretion over punishment should be vigilant about how the situational cues may be affecting their psychological reactions to the transgressors and ultimately, their punishment decisions.

## Author Contributions

The authors contributed equally to this work. LP collected, analyzed, and wrote up the field data. LP and CM conceived the experiments. CM ran the experiments, analyzed and wrote up the lab data. LP and CM discussed the results and implications and commented on the manuscript at all stages, and wrote the paper jointly.

## Conflict of Interest Statement

The authors declare that the research was conducted in the absence of any commercial or financial relationships that could be construed as a potential conflict of interest.

## Footnotes

1 http://www.mirror.co.uk/money/free-birthday-offers-deals-club-5260023

2 http://www.tmz.com/2012/12/02/game-lapd-birthday-bentley-registration/

3 We use this terminology to indicate that such drivers are at the *per se* margin, not to understate the danger or seriousness of driving at these BAC levels. Driving with a marginal BAC of 0.07, for example, still elevates the risk of injury and fatality considerably, and we intend no judgment on the ethicality (or lack thereof) of such behavior.
